# Relationship between cervical posterior subcutaneous fat tissue thickness and the presence and degree of cervical intervertebral disc degeneration

**DOI:** 10.1097/MD.0000000000029890

**Published:** 2022-07-15

**Authors:** Yavuz Yuksel, Tarkan Ergun, Ebru Torun

**Affiliations:** a Department of Radiology, Faculty of Medicine, Alaaddin Keykubat University, Alanya, Turkey.

## Abstract

**Objective:**

This study aimed to investigate the relationship between cervical region subcutaneous fat tissue thickness and the presence and level of cervical intervertebral disc degeneration (IVDD).

**Methods:**

Magnetic resonance imaging examinations of patients referred to our clinic for the investigation of neck pain were evaluated retrospectively. A total of 300 women aged 30–40 years were included in the study. The presence and level of IVDD were evaluated for each patient. The cervical subcutaneous fat tissue thickness was also measured.

**Results:**

IVDD was determined as Grade 1 for 88 patients (29.3%), Grade 2 for 56 patients (18.6%), Grade 3 for 82 patients (27.3%), Grade 4 for 60 patients (20%), and Grade 5 for 14 patients (4.6%). Subcutaneous fat tissue thickness was higher in patients with cervical disc degeneration (mean: 6.28 ± 0.19 mm) than in those without cervical disc degeneration (mean: 5.33 ± 0.18 mm) (*P* = .001). There was a positive correlation between the degree of cervical disc degeneration and subcutaneous fat tissue thickness ( = 0.001, *r* = 0.245).

**Conclusion:**

An increase in the cervical fat tissue thickness is a predisposing factor for the development of degeneration of the intervertebral disc. There is a close relationship between subcutaneous fat tissue thickness and the degree of degeneration.

## 1. Introduction

Cervical intervertebral disc degeneration (IVDD) causes neck pain and upper extremity numbness and is the main cause of spinal complaints. The incidence of cervical degenerative diseases increases with age.^[1]^ Cervical IVDD is defined as a cell-mediated abnormal response to progressive structural deficiency of the discs.^[2]^

As late-stage IVDD progresses to disc and cervical spinal canal stenosis, it can require surgical treatment and result in a heavy financial burden. Determining the predisposing factors of IVDD is, therefore, important for early diagnosis and treatment.

The relationship among IVDD and obesity, genetic factors, trauma, smoking, sagittal vertebral column morphology, age, sex, and ethnic group has previously been investigated.^[3–13]^ Obesity is a serious health problem with increasing global incidence and has been defined as a risk factor for lumbar IVDD.^[3]^ Body mass index (BMI) is generally used as an indicator for the measurement of fat tissue, but the general type of body fat storage varies with each person. Therefore, BMI cannot accurately measure local obesity around the cervical region.

To the best of our knowledge, there is no other study investigating the relationship between cervical region subcutaneous fat tissue thickness and the presence of cervical IVDD.

Subcutaneous fat tissue measurement is a simple method that provides information on the fat quantity and distribution in humans. Measurements obtained from skin fold regions have been reported to be strong indicators of morbidity.^[14–16]^

Magnetic resonance imaging (MRI) is considered the best noninvasive method to study and evaluate IVDD in human and animal models as it reflects both the macromolecule concentration and structural integrity of the intervertebral disc.^[17,18]^

This study aimed to investigate the relationship between cervical region subcutaneous fat tissue thickness and the presence and level of cervical IVDD.

## 2. Material and Method

Cervical MRI results of 1760 patients who were referred to our clinic between 2019 and 2020 for the investigation of neck pain were evaluated retrospectively. Patients with a cervical mass, cervical congenital anomaly, cervical vertebral fracture, discitis, osteomyelitis, spondylolysis, and spondylolisthesis, and those with complaints other than neck pain, or those with a history of cervical surgery were not included in the study. To eliminate age and sex differences that could create a trend toward degeneration development, a study group was established with women aged 30–40 years. A total of 300 female patients with a mean age of 35.2 ± 2.9 years were evaluated within the scope of the study.

The approval of the Ethics Committee was not obtained as the study was retrospective and involved only noninvasive procedures.

All examinations were conducted with a 1.5 tesla 16-coil MRI device (Signa: GE Medical Systems, Milwaukee, WI). The sagittal T1-weighted images (WI), sagittal T2-WI, and axial T2-weighted fast spin-echo images were obtained with the imaging protocol used (T1WI: repetition time/echo time 520/12 ms, echo train length 4; T2WI: 5000/102 ms, echo train length 16; slice thickness 5 mm, no slice gap, field of view 24 cm for sagittal images and 16 cm for axial images, matrix 256 × 192, 4 excitations).

**Figure 1.**
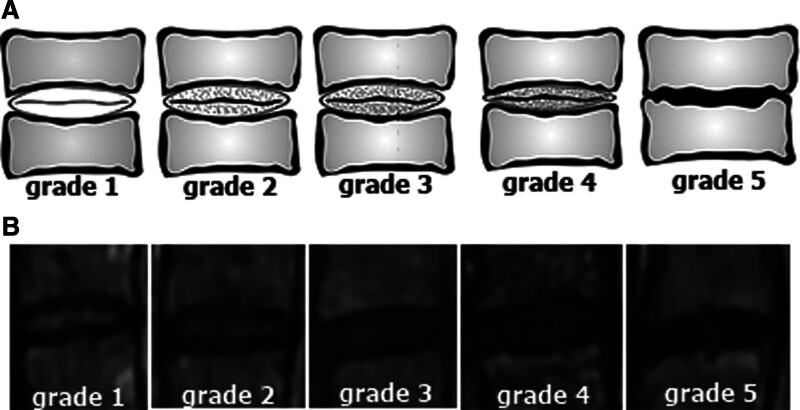
Intervertebral disc degeneration level. The illustration (A) and magnetic resonance images (B) of the degree of cervical intervertebral disc degeneration.

The presence and degree of cervical IVDD were evaluated in all patients. Evaluation of the degree of cervical IVDD was through the consensus of two radiologists specializing in neuroradiology interpreting the sagittal T2-WI, and classification was according to the simple and reproducible grading system defined by Miyazaki et al^[19]^ (Table 1 and Fig. 1A and B).

**Table 1:** Grading system for cervical intervertebral disc degeneration.

Grade	Nucleus signal intensity and nucleus structure	Distinction of nucleus and annulus	Disc height
1	Hyperintense homogenous, white	Clear	Normal
2	Hyperintense inhomogenous with horizontal band, white	Clear	Normal
3	Intermediate inhomogenous, gray to black	Unclear	Normal to decreased
4	Hypointense inhomogenous, gray to black	Lost	Normal to decreased
5	Hypointense inhomogenous, gray to black	Lost	Collapsed

**Figure 2.**
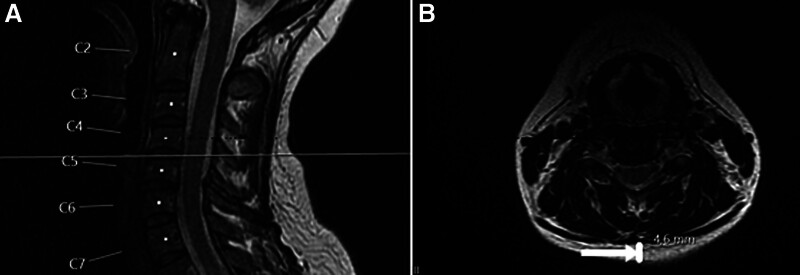
A 34-yr-old woman with neck pain. Cervical midsagittal T2-weighted image (A) and measurement of cervical subcutaneous fat tissue thickness in the axial T2 fast spin-echo image (B) passing through the C4-C5 intervertebral disc space (arrow).

In addition, the C4-C5 intervertebral disc space was determined as the reference region for subcutaneous fat tissue thickness measurement. The C4-C5 level was chosen to prevent measurement errors since the angle of the axial image passing through the intervertebral disc level at other levels with the ground plane can vary. The subcutaneous fat tissue thickness was measured in mm (millimeter) from the midaxial posterior cut in the axial T2-WI passing through the C4-C5 intervertebral disc level (Fig. 2A and B). All evaluations were made on the workstation, and measurements were made electronically.

In addition, the data of both groups (with and without disc degeneration) were compared with the *t* test. The relationship between the degree of IVDD and subcutaneous fat tissue thickness values was evaluated with the Mann–Whitney *U* test. A *P* value of <.05 was considered statistically significant. Statistical analysis was performed using the statistical package for social sciences 22.0 (SPSS Inc., Chicago, IL) package program.

## 3. Results

We found 88 (29.3%) of the 300 women to be Grade 1, and these patients were accepted as the group without disc degeneration. IVDD was Grade 2 in 56 patients (18.6%), Grade 3 in 82 patients (27.3%), Grade 4 in 60 patients (20%), and Grade 5 in 14 patients (4.6%). The subcutaneous fat tissue thickness was higher in the participants with cervical disc degeneration (mean: 6.28 ± 0.19 mm) than in those without cervical disc degeneration (mean: 5.33 ± 0.18 mm), and this difference was statistically significant (*P* = .001).

**Figure 3.**
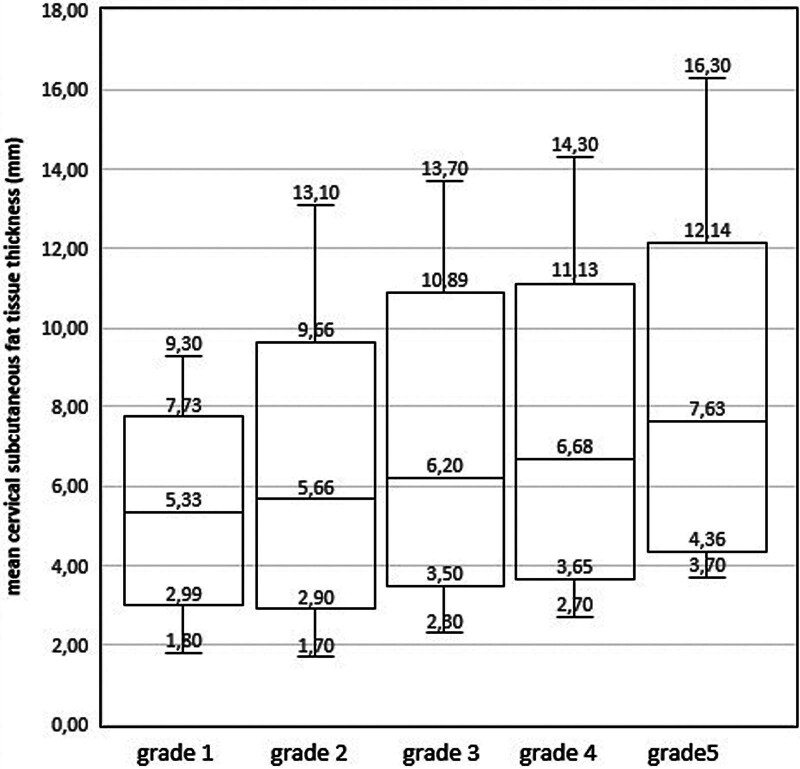
Box plot of the distribution of subcutaneous fat tissue thickness according to the intervertebral disc degeneration levels.

The mean cervical subcutaneous fat tissue thickness at the C4-C5 intervertebral disc level was 5.33 ± 0.18 mm in the Grade 1 group, 5.66 ± 0.30 mm in the Grade 2 group, 6.20 ± 0.27 mm in the Grade 3 group, 6.68 ± 0.35 mm in the Grade 4 group, and 7.63 ± 1.09 mm in the Grade 5 group (Fig. 3). A positive correlation was found between the degree of IVDD and the subcutaneous fat tissue thickness (*P* = .001, *r* = 0.245).

## 4. Discussion

To the best of our knowledge, this study is the first to examine the relationship between the presence and degree of cervical IVDD and the cervical subcutaneous fat tissue thickness. We found a close relationship between the presence and degree of IVDD and increased subcutaneous fat tissue thickness.

The relationship between cervical degenerative changes and age has been investigated in many studies. Matsumoto et al^[20]^ evaluated the cervical MR images of 497 participants and found the incidence of cervical degenerative changes to increase with age. In addition, cervical degenerative changes may vary with sex. Wang et al^[21]^ found cervical degenerative changes to be more common in women. We selected a very specific sample group to eliminate sex and age-related differences in this study, and only women aged 30–40 years were included.

Although a consensus on the pathophysiological mechanism causing cervical degenerative disc disease has not been elucidated, there are several assumptions. The disorder is considered a normal part of aging according to the current approach, but some jobs, repetitive movements, and other causes such as trauma can speed up the process.^[22]^ Kok et al^[23]^ have reported a close relationship between the subcutaneous fat tissue thickness around the knee joint and patellar chondromalacia. They stated that the mechanical effect caused by the increased subcutaneous fat tissue thickness around the knee joint results in increased development and degree of patellar chondromalacia.^[23]^

Our study’s contribution to the literature is finding a close relationship between the degree of IVDD and subcutaneous fat tissue thickness (*P* = .001, *r* = 0.245). Therefore, increased subcutaneous fat tissue thickness may cause increasing recurrent microtrauma of the disc.

Fat tissue, previously thought to be an energy store, is now accepted as an endocrine organ that secretes cytokines such as tumor necrosis factor (TNF) and interleukin-1 (IL-1), in addition to adipokines such as leptin, adiponectin, and resistin. It has been shown to play a key role in cartilage destruction, especially with IL-1 and TNF.^[24–26]^

Intervertebral discs are surrounded by endplates in both the cranial and caudal aspects consisting of bone and hyaline cartilage.^[27]^ An increase in subcutaneous fat tissue thickness in the cervical region causes cytokines such as IL-1 and TNF that play a role in cartilage destruction to be released from the fat tissue and to be formed in the cervical vertebral endplates and fibrocartilage-like intervertebral discs. This can lead to secondary adverse effects by causing disrupted diffusion into the cells inside the intervertebral disc, loss of hydration in the disc, and reduced disc load-bearing capacity.

The limitations of our study include the lack of a history of smoking and sports activities, medical information, and physical measurements (height and kilograms), in addition to the inability to calculate the BMI.

## 5. Conclusions

Despite these limitations, our study, which determined a positive correlation between an increase in the thickness of cervical subcutaneous fat tissue and the presence and degree of cervical IVDD, is important in terms of revealing that an increase in cervical fat tissue thickness is an association event that may be a predisposing factor for the development of cervical IVDD.

## Author contributions

Conceptualization: Yavuz Yuksel and Tarkan Ergun

Data curation: Yavuz Yuksel, Tarkan Ergun, and Ebru Torun

Formal analysis: Tarkan Ergun and Yavuz Yuksel

Investigation: Yavuz Yuksel and Ebru Torun

Methodology: Yavuz Yuksel

Resources: Yavuz Yuksel, Tarkan Ergun, and Ebru Torun

Software: Yavuz Yuksel

Supervision: Yavuz Yuksel

Writing—original draft: Yavuz Yuksel and Tarkan Ergun

Writing—review and editing: Yavuz Yuksel, Tarkan Ergun, and Ebru Torun
